# Combined associations of obesity and physical activity with pain, fatigue, stiffness and anxiety in adults with spondyloarthropathies: UK Biobank study

**DOI:** 10.1093/rap/rkae109

**Published:** 2024-09-03

**Authors:** Matthew J Roberts, William Johnson, Sepehr Qooja, Arumugam Moorthy, Nicolette C Bishop

**Affiliations:** National Centre for Sport and Exercise Medicine, School of Sport, Exercise and Health Sciences, Loughborough University, Loughborough, UK; National Institute for Health Research (NIHR) Leicester Biomedical Research Centre, University Hospitals of Leicester, National Health Service (NHS) Trust and the University of Leicester, Leicester, UK; National Centre for Sport and Exercise Medicine, School of Sport, Exercise and Health Sciences, Loughborough University, Loughborough, UK; National Institute for Health Research (NIHR) Leicester Biomedical Research Centre, University Hospitals of Leicester, National Health Service (NHS) Trust and the University of Leicester, Leicester, UK; National Centre for Sport and Exercise Medicine, School of Sport, Exercise and Health Sciences, Loughborough University, Loughborough, UK; National Institute for Health Research (NIHR) Leicester Biomedical Research Centre, University Hospitals of Leicester, National Health Service (NHS) Trust and the University of Leicester, Leicester, UK; National Centre for Sport and Exercise Medicine, School of Sport, Exercise and Health Sciences, Loughborough University, Loughborough, UK; Department of Rheumatology, University Hospitals of NHS Trust, College of Life Sciences, University of Leicester, Leicester, UK; National Centre for Sport and Exercise Medicine, School of Sport, Exercise and Health Sciences, Loughborough University, Loughborough, UK; National Institute for Health Research (NIHR) Leicester Biomedical Research Centre, University Hospitals of Leicester, National Health Service (NHS) Trust and the University of Leicester, Leicester, UK

**Keywords:** obesity, physical activity, inflammatory spondyloarthropathies, pain, fatigue, anxiety, mobility

## Abstract

**Objective:**

Inflammatory spondyloarthropathies are associated with pain, fatigue, stiffness and anxiety. The National Institute for Health and Care Excellence and the EULAR provide limited lifestyle guidance for managing symptoms with inflammatory spondyloarthropathies. We investigated the combined associations of obesity and physical activity with symptom severity in inflammatory spondyloarthropathies.

**Methods:**

The relationship between BMI, physical activity and symptom severity (spinal and general pain, fatigue, anxiety, mobility) was examined in people with ISpAs (*n* = 1577). BMI categories were normal weight (18.5–24.9 kg/m^2^), overweight (25.0–29.9 kg/m^2^) and obese (≥30 kg/m^2^). Physical activity was assessed via the International Physical Activity Questionnaire (low < 600 metabolic equivalent of task (MET)-min/week, moderate ≥ 600 METs, high ≥ 3000 METs). Statistical models adjusted for confounders, including medication, estimated the likelihood (odds ratios, OR) of higher symptom severity across BMI and physical activity categories.

**Results:**

Overweight and obesity, compared with normal weight, were linked to higher severity of all symptoms, with stronger associations for obesity (OR ≥ 2.34, *P* < 0.001) than overweight (OR ≥ 1.37, *P* ≤ 0.032). Moderate activity, compared with low, was associated with lower severity of all symptoms (OR ≤ 0.77, *P* ≤ 0.032). High activity, compared with low, was associated with lower severity of fatigue, anxiety and mobility issues (OR ≤ 0.74, *P* ≤ 0.029), but associations with spinal and general pain were not significant (OR ≤ 0.80, *P* ≥ 0.056). No BMI-by-physical activity combinations were detected, indicating physical activity benefits all BMI groups to a similar extent.

**Conclusion:**

National Institute for Health and Care Excellence and EULAR guidance for inflammatory spondyloarthropathies should emphasize maintaining a normal weight. Moderate physical activity is optimal for reducing symptom severity and should be promoted in lifestyle guidance.

## Graphical Abstract

**Graphical Abstract:**
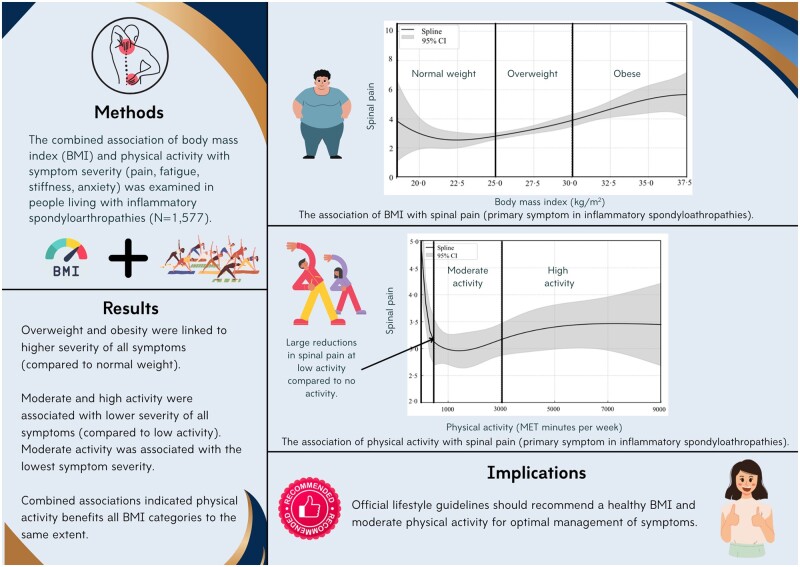

Key messages

- Overweight and obesity are linked to higher symptom severity in inflammatory spondyloarthropathies.
- Physical activity benefits all BMI groups; moderate activity is associated with lowest symptom severity.
- Even low physical activity is associated with lower spinal pain (compared to complete inactivity).

## Introduction

Inflammatory spondyloarthropathies encompass a group of chronic inflammatory diseases that primarily affect the spine and major joints [1]. Inflammatory spondyloarthropathies manifest through inflammation of joints, ligaments and tendons, leading to common symptoms such as spinal and general pain, fatigue, stiffness and reduced mobility [1]. These primary symptoms can contribute to social withdrawal, diminished quality of life and heightened levels of anxiety and depression [2].

The World Health Organization advocates for all adults to be physically active and have a healthy body weight, as both physical inactivity and obesity are associated with preventable health issues, including cardiovascular disease and other non-communicable diseases [3]. Regular physical activity not only aids the management of inflammation in both healthy individuals and those with chronic inflammatory diseases, but also helps alleviate pain, fatigue and poor mental health, independent of weight loss [4, 5]. Conversely, obesity is linked with chronic low-grade inflammation, fatigue, heightened levels of stress, anxiety and depression [6]. The excess weight also adds mechanical stress to joints, potentially exacerbating pain [7].

Despite the acknowledged benefits of physical activity and weight management in other populations, disease management guidance for those living with inflammatory spondyloarthropathies is dominated by pharmaceutical therapies [8]. Disease management typically involves progressing through stronger and relatively expensive medications with some lifestyle modification [9]. Smoking cessation is commonly advised owing to the strong association with inflammation [9]. However, despite people with inflammatory spondyloarthropathies having a positive attitude towards physical activity and other lifestyle modifications for disease management [10], official guidance is vague.

Systematic reviews and meta-analyses document an association between elevated BMI and poor symptom management in populations with inflammatory diseases such as rheumatoid arthritis and osteoarthritis [11, 12]. However, lifestyle recommendations for people with rheumatic diseases highlighted that most research on the effect of obesity on disease activity has focused on rheumatoid and osteoarthritis, with limited studies on inflammatory spondyloarthritis [13]. This distinction is crucial because the associations may differ due to the unique joint involvement, underlying mechanisms, and symptoms in inflammatory spondyloarthropathies. For instance, major symptoms of inflammatory spondyloarthropathies include back pain, fatigue, enthesitis and sacroiliitis [1], whereas rheumatoid arthritis typically affects small joints with symmetrical involvement and localized joint pain [14].

The National Institute for Health and Care Excellence (NICE) has no guidance on body weight management for inflammatory spondyloarthritis and offers very brief guidance on non-pharmacological management techniques, simply saying that people with inflammatory spondyloarthritis should be referred to a physiotherapist and should consider hydrotherapy [15]. The EULAR has intermittently addressed physical activity but has rarely acknowledged body weight management in official guidance. Further, guidance is limited to all rheumatic diseases, rather than specific guidelines for inflammatory spondyloarthropathies. In 2018, EULAR stated that physical activity has health benefits for people with rheumatic diseases and should be planned with a health care professional, but there was no mention of body weight [16]. In 2021, EULAR simply recommended promoting physical activity and controlling body weight to manage inflammation and cardiovascular disease risk [17]. In 2022, EULAR again recommended for physical activity to be encouraged, but did not expand on this due to the heterogeneity of studies, and there was no mention of body weight management [8]. The guidance also called for research on potential harms of excessive exercise. In 2023, EULAR published recommendations for the management of fatigue in people with inflammatory rheumatic diseases and musculoskeletal diseases. It was highlighted that fatigue is still present even with low disease activity and set a research priority for understanding the contributions to fatigue, which may include sleep, physical activity, cognitive behaviour management, and body weight management [18].

The aim of our study was to investigate the association of obesity and physical activity with symptom severity in people living with inflammatory spondyloarthritis.

## Methods

### Study

We used data from the UK Biobank, a prospective population-based cohort study that recruited over 500 000 adults aged 40–69 years between 2006 and 2010 [19]. Ethical approval was granted by the Northwest Multi-Centre Research Ethics Committee (Ref: 11/NW/0382), and all participants provided written informed consent. The current study was conducted as part of the UK Biobank approved project 80843.

### Sample

**Figure 1.**
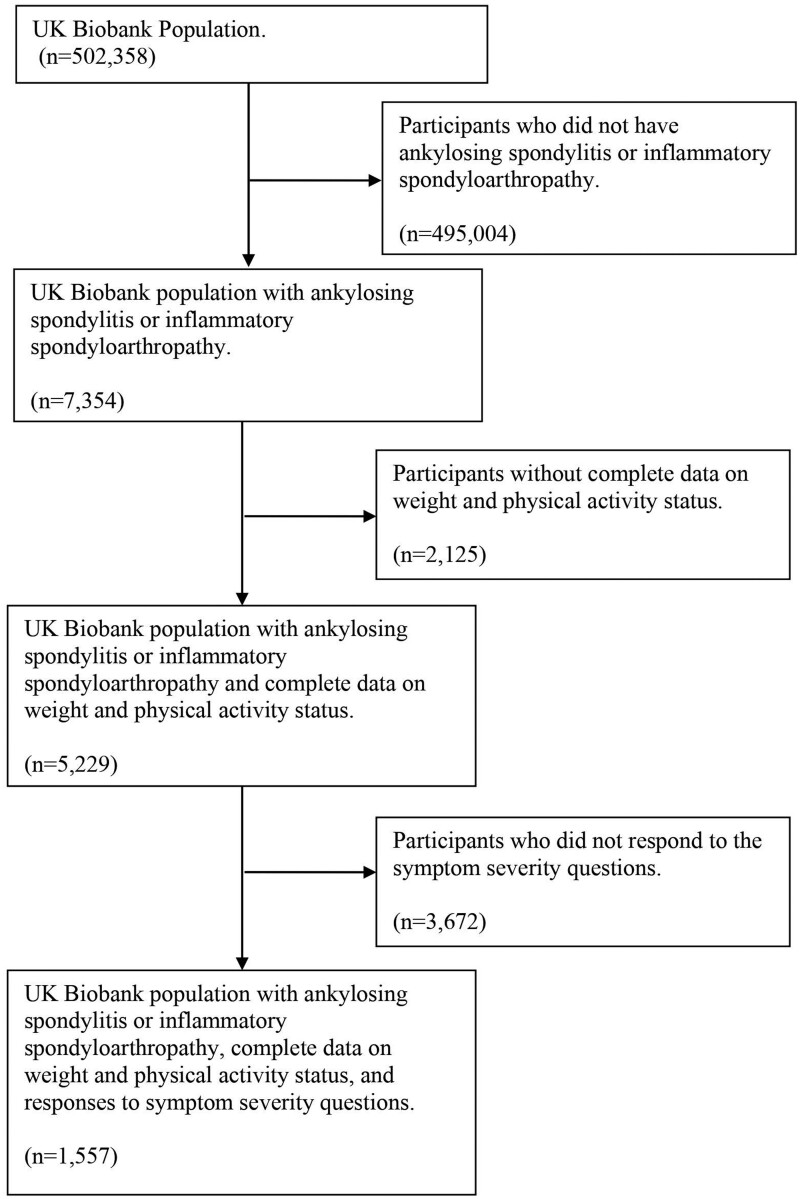
Participant flow diagram

The sample comprised 7354 individuals (53.4% female) with either ankylosing spondylitis (31.4%) or another inflammatory spondyloarthropathy (68.6%). Cases were identified by UK Biobank through primary care records, hospital admissions and self-report using ICD-10 codes. Individuals listed in death registers were excluded from the analysis as their characteristics may have differed, leading to inaccurate or misleading conclusions. A sample selection flow diagram is shown in Fig. 1.

### Outcomes: symptom severity

A rating of spinal pain was only available for individuals who reported pain for more than three months (data code: 120019) and who did not experience pain or discomfort all over the body (data code: 120021). Specifically, individuals were asked if they were troubled by pain or discomfort that has been present for more than three months (date code: 120019; yes, no, do not know). Those who said yes progressed to data code 120021 which asked individuals whether they had experienced pain or discomfort all over the body (yes, no, do not know). Those responding no were then asked whether they had experienced pain or discomfort in their back over the past three months, and if so, to rate the pain on a scale of 0–10 (0 = no pain, 10 = pain as bad as it could be). Fatigue severity (data code: 120040) asked participants to rate their fatigue level over the past week using the options ‘none, slight, moderate, or severe’. Mobility problems (data code: 120098), general pain or discomfort (data code: 120101) and anxiety or depression (data code: 120102) asked participants to describe their symptom severity on the assessment day (none, slight, moderate, severe, extreme).

### Exposure: weight status

Height was measured using a Seca 240 cm stadiometer and weight was measured using a Tanita BC418MA body composition analyzer. BMI was computed as kg/m^2^ and categorized as normal weight (18.5–24.9 kg/m^2^), overweight (25.0–29.9 kg/m^2^), or obese (≥30 kg/m^2^).

### Exposure: physical activity

Physical activity was evaluated using the short-form International Physical Activity Questionnaire (IPAQ). Based on their metabolic equivalents of task min (METs) per week for both walking and moderate-to-vigorous physical activities, participants were categorized as having either low (<600 METs per week), moderate (600–2999 METs per week), or high (≥3000 METS per week) physical activity [20]. Low activity is equivalent to less than 3 h of brisk walking per week [20].

### Potential confounders

Chronological age in decimal years was determined from date of birth and date of attending a UK Biobank assessment centre. Sex was obtained from local NHS Primary Care Trust registers at recruitment. Ethnicity, smoking status, alcohol intake frequency, use of medication for pain relief, disease duration and the Townsend Deprivation Index were assessed through touch-screen questionnaires.

### Statistical analysis

Descriptive statistics were produced for each weight status and physical activity group.

Generalized linear models were used to investigate the associations of BMI and physical activity category with each symptom severity outcome. Ordinal logistic regression was used for spinal pain (ordinal: 0–10 score) and other outcomes (ordinal: none, slight, moderate, extreme or severe). Models were adjusted for age, sex and ethnicity. Further models were developed to adjust for age, sex, ethnicity, disease duration, alcohol intake, smoking status, medication and deprivation. All estimates are presented as odds ratios (95% confidence intervals).

Eight models were constructed for each symptom severity marker. Model one included BMI as the independent variable, model two included physical activity as the independent variable, model three included both BMI and physical activity and model four included both, along with the interaction term. Models were then repeated to include the additional covariates previously mentioned. Proportional odds were assessed for models, indicating the assumption was met for all models (*P* > 0.05). Likelihood ratio tests were performed to determine whether the interaction between BMI and physical activity significantly improved the model fit (model three vs model four). This indicated that adding an interaction did not improve the model fit (*P* > 0.05) and model three was used as the primary model. All models are presented in the Supplementary Material, available at *Rheumatology Advances in Practice* online.

In addition to examining BMI category, we fit models integrating BMI (kg/m^2^) and physical activity (MET min per week) as continuous variables on restrictive cubic spline models for spinal pain as this is the major symptom of inflammatory spondyloarthropathies. Restricted cubic splines are a common approach used to model the non-linear relationship between an exposure and an outcome [21]. Figures were produced to illustrate the results.

## Results

Participant characteristics split by BMI and IPAQ are presented in Table 1. Characteristics for each individual group (i.e. normal weight, low activity) are presented in Supplementary Table S1, available at *Rheumatology Advances in Practice* online. More females than males were normal weight. Obesity and low physical activity were associated with a lower prevalence of being a never-smoker and a higher prevalence of taking medication for pain relief.

**Table 1.** Descriptive statistics for each weight status group and each physical activity group.

		Weight status			Physical activity		
	·	Normal weight	Overweight	Obese	Low	Moderate	High
		(*N* = 511)	(*N* = 629)	(*N* = 437)	(*N* = 330)	(*N* = 623)	(*N* = 624)
Age (years)	Median (IQR)	57 (52, 63)	58 (53, 64)	59 (53, 63)	58 (52, 62)	59 (53, 63)	59 (53, 64)
Disease duration (years)	Median (IQR)	11.5 (5.8, 23.3)	12.5 (5.6, 25.0)	9.4 (4.8, 19.1)	12.0 (6.3, 24.5)	12.3 (5.6, 23.3)	10.5 (4.8, 20.8)
Sex							
Male	*N* (%)	183 (35.8)	327 (52.0)	193 (44.2)	141 (42.7)	273 (43.8)	289 (46.3)
Female	*N* (%)	328 (64.2)	302 (48.0)	244 (55.8)	189 (57.3)	350 (56.2)	335 (53.7)
Ethnicity							
White	*N* (%)	485 (94.9)	615 (97.8)	429 (98.2)	313 (94.8)	608 (97.6)	608 (97.4)
South Asian	*N* (%)	4 (0.8)	7 (1.1)	4 (0.9)	6 (1.8)	4 (0.6)	5 (0.8)
Black	*N* (%)	2 (0.4)	5 (0.8)	2 (0.5)	3 (0.9)	4 (0.6)	2 (0.3)
Mixed/other	*N* (%)	20 (3.9)	2 (0.3)	2 (0.5)	8 (2.4)	7 (1.1)	9 (1.4)
Smoking status							
Never	*N* (%)	273 (53.3)	321 (51.0)	189 (43.2)	153 (46.4)	315 (50.6)	315 (50.5)
Previous	*N* (%)	190 (37.3)	259 (41.2)	208 (47.6)	131 (39.7)	258 (41.4)	268 (42.9)
Current	*N* (%)	48 (9.4)	49 (7.8)	40 (9.2)	46 (13.9)	50 (8.0)	41 (6.6)
Alcohol intake							
Daily or almost daily	*N* (%)	132 (25.8)	144 (22.9)	86 (19.7)	67 (20.3)	151 (24.2)	143 (22.9)
Three or four times a week	*N* (%)	127 (24.9)	155 (24.6)	85 (19.5)	69 (20.9)	141 (22.6)	157 (25.2)
Once or twice a week	*N* (%)	123 (24.1)	163 (25.9)	92 (21.1)	76 (23.0)	157 (25.2)	145 (23.2)
One to three times a month	*N* (%)	57 (11.2)	69 (11.0)	55 (12.6)	37 (11.2)	77 (12.4)	68 (10.9)
Special occasions	*N* (%)	44 (8.6)	61 (9.7)	85 (19.5)	53 (16.1)	63 (10.1)	74 (11.9)
Never	*N* (%)	28 (5.5)	37 (5.9)	34 (7.8)	28 (8.5)	34 (5.5)	37 (5.9)
Townsend Deprivation Index	Median (IQR)	−2.71 (−3.95, −0.72)	−2.50 (−3.83, −0.17)	−1.79 (−3.53, 0.95)	−2.36 (−3.73, 0.57)	−2.45 (−3.88, −0.34)	−2.38 (−3.76, −0.15)
Medication for pain relief							
No medication	*N* (%)	253 (49.5)	286 (45.4)	152 (34.8)	129 (39.2)	281 (45.1)	280 (44.9)
Pain relief and anti-inflammatory drugs (e.g. Aspirin, Ibuprofen, Paracetamol)	*N* (%)	230 (45.0)	300 (47.7)	249 (56.9)	176 (53.7)	297 (47.6)	305 (48.9)
Gastrointestinal medication (e.g. Ranitidine and Omeprazole)	*N* (%)	21 (4.1)	37 (5.9)	31 (7.1)	16 (4.9)	38 (6.1)	35 (5.6)
Bowel regulation (e.g. Laxatives)	*N* (%)	7 (1.4)	6 (1.0)	5 (1.1)	7 (2.2)	7 (1.1)	4 (0.6)

### The effect of BMI on symptom severity

**Figure 2.**
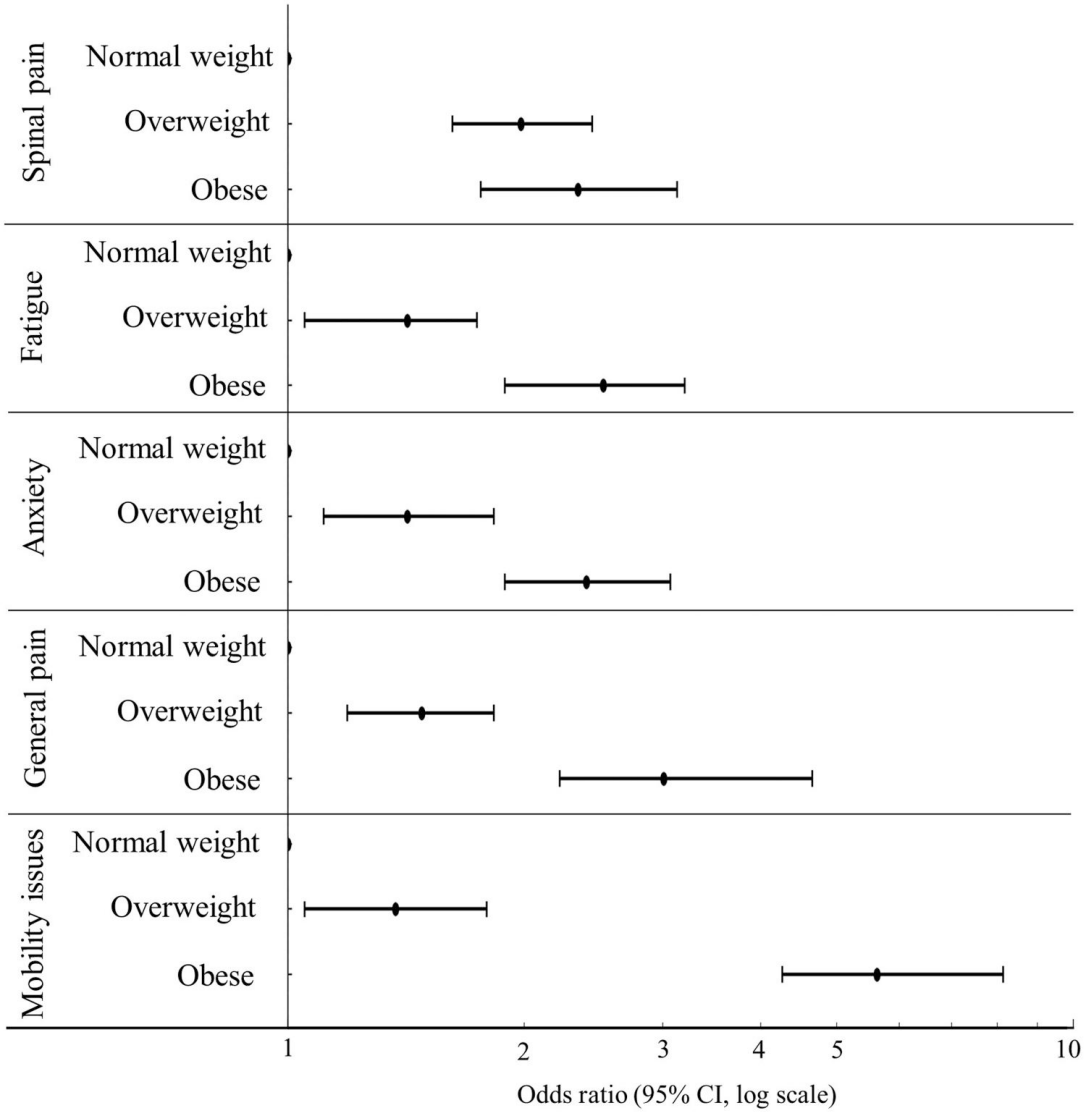
Association of body mass index category with symptom severity indicators

**Figure 3.**
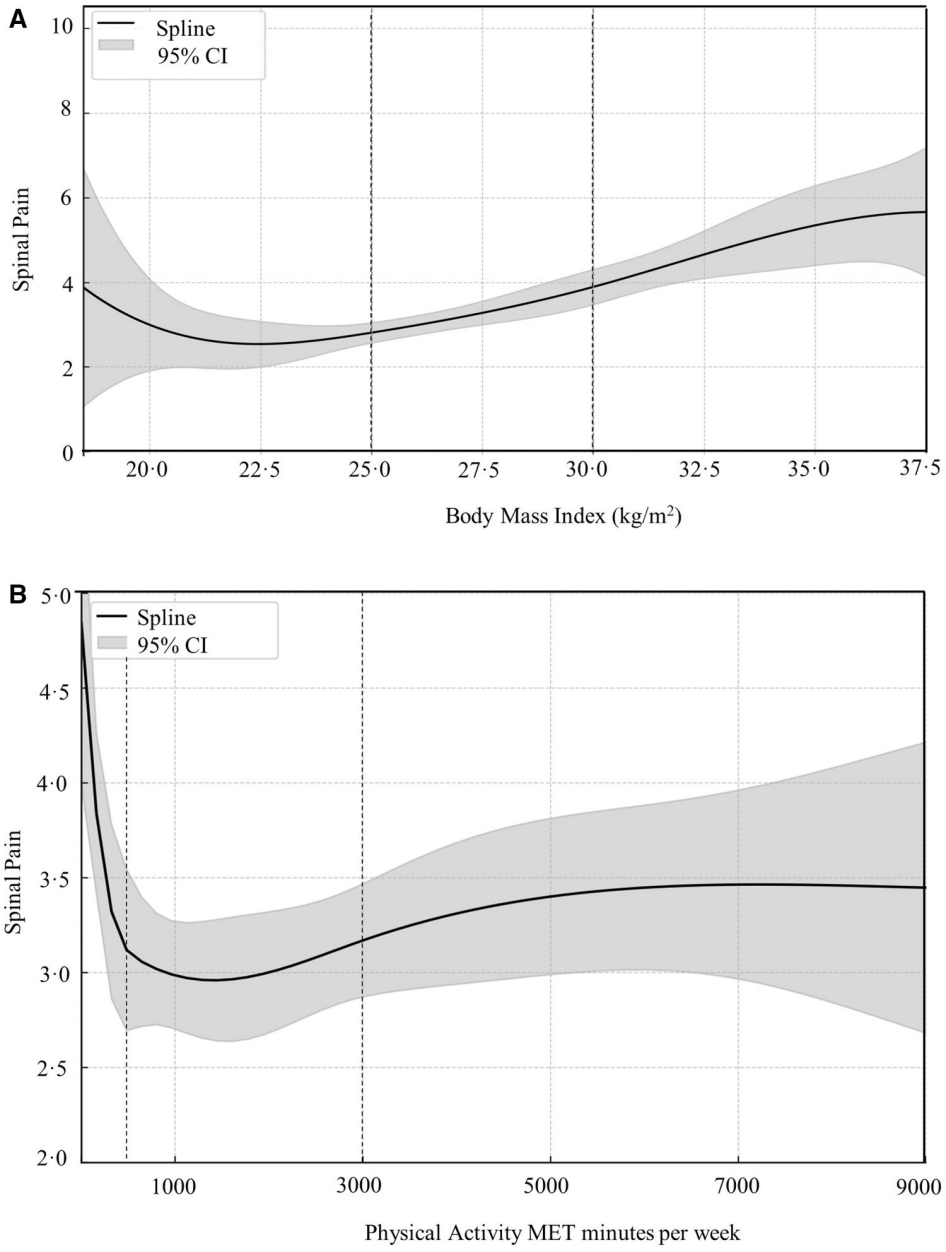
The relationship between body mass index (panel A) and physical activity (panel B) with spinal pain. Dashed lines at 25.0 and 30.0 on panel (A) demonstrate the starting point of overweight and obesity, respectively. Dashed lines at 600 and 3000 on panel (B) demonstrate the starting point of moderate and high activity, respectively. MET, metabolic equivalent of task. Spinal pain is rated from 0 to 10 (0 = no pain, 10 = pain as bad as it could be)

Overweight and obesity exhibited a positive association with all indicators of symptom severity compared with normal weight (Fig. 2, Supplementary Tables S2–S6, available at *Rheumatology Advances in Practice* online). Individuals with overweight were more likely to experience higher spinal pain, fatigue, anxiety and depression, general pain and mobility issues relative to those of normal weight (Fig. 2). The association was even more pronounced in individuals with obesity, who showed an increased likelihood of higher symptom severity than people with overweight compared with individuals of normal weight (Fig. 2). The lowest spinal pain was seen in those with a BMI of 22.5 kg/m^2^, after which there was a positive association between BMI and spinal pain (Fig. 3). Model estimates did not significantly change from baseline models (adjusted for age, sex and ethnicity) after further adjustment for alcohol intake, smoking status, pain relief medication, socioeconomic deprivation and disease duration (Supplementary Tables S7–S11, available at *Rheumatology Advances in Practice* online).

### The effect of physical activity on symptom severity

**Figure 4.**
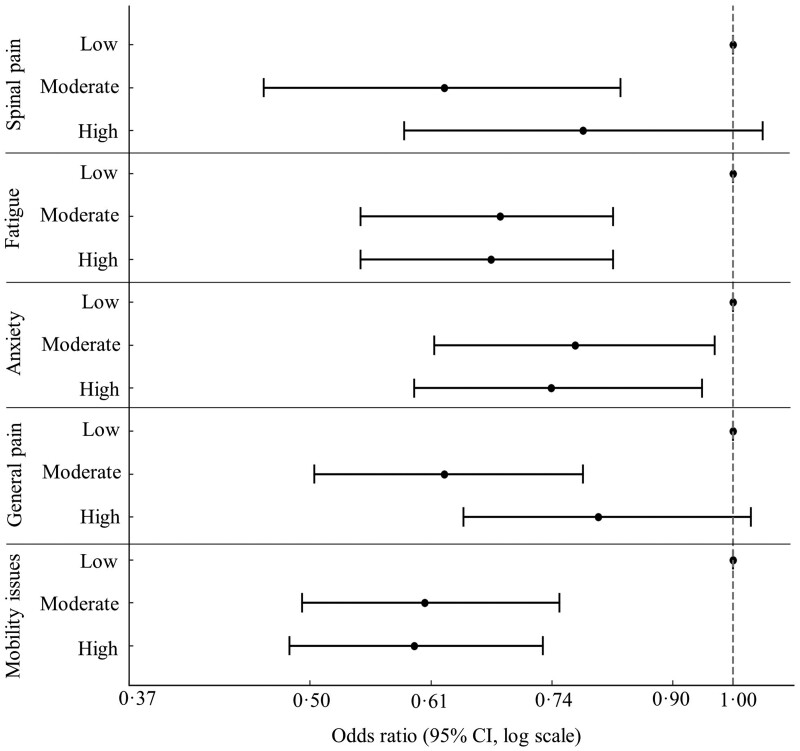
Association of physical activity level with symptom severity indicators

Moderate and high physical activity exhibited a negative association with all indicators of symptom severity compared with low activity (Fig. 4, Supplementary Tables S2–S6, available at *Rheumatology Advances in Practice* online). Both moderate and high levels of physical activity were associated with significant and similarly better fatigue, mobility and anxiety compared with low activity levels. However, moderate physical activity had a stronger negative association than high activity with spinal and general pain compared with low physical activity levels. Although high activity was associated with lower severity of spinal and general pain compared with low activity, the negative association was not statistically significant. Spline modelling suggested the lowest spinal pain was seen in those with moderate activity, but a significant reduction in spinal pain was seen between those doing no activity (zero METS per week) and those doing a small amount of physical activity (100–500 METS per week) (Fig. 3). Model estimates did not significantly change from baseline models (adjusted for age, sex and ethnicity) after further adjustment for alcohol intake, smoking status, pain relief medication, socioeconomic deprivation, and disease duration (Supplementary Tables S7–S11, available at *Rheumatology Advances in Practice* online).

There were no BMI-by-physical activity (combined association) interactions suggesting the benefits of physical activity were similar across BMI categories (Supplementary Tables S2–S11, available at *Rheumatology Advances in Practice* online).

## Discussion

The findings indicate that prioritizing a normal BMI is crucial for the lifestyle management of people living with inflammatory spondyloarthritis. A normal BMI was linked to better management of spinal pain, fatigue, anxiety, general pain and mobility compared with those with overweight or obesity. Encouraging moderate physical activity is also essential, as it was associated with lower symptom severity across all symptoms compared with low activity levels. The positive impact of physical activity on symptom severity was consistent across BMI categories. Consequently, NICE and EULAR guidelines for symptom management should emphasize the importance of achieving a normal BMI and engaging in moderate physical activity for individuals with inflammatory spondyloarthropathies. It is worth noting that reverse causality is a limitation of the current study, and longitudinal studies are needed to confirm the findings. For example, are people with low physical activity less active because of pain, or are they less active, leading to higher pain?

Systematic reviews and meta-analyses have documented an association between elevated BMI and poor symptom management in populations with inflammatory diseases, such as rheumatoid arthritis and osteoarthritis [11, 12]. However, the 2021 EULAR lifestyle recommendations for people with rheumatic diseases highlighted that most research on the effect of obesity on disease activity has focused on rheumatoid and osteoarthritis, with limited studies on inflammatory spondyloarthritis [13]. This distinction is crucial because the associations may differ due to the unique joint involvement, underlying mechanisms, and symptoms in inflammatory spondyloarhropathies. For instance, major symptoms of inflammatory spondyloathropathies include back pain, fatigue, enthesitis and sacroiliitis [1], whereas rheumatoid arthritis typically affects small joints with symmetrical involvement and localised joint pain [14].

In people with ankylosing spondylitis, previous research has focused on the relationship between body weight and disease outcomes in response to tumor necrosis factor-alpha inhibitors [13], but this limits the study population to those with sufficient disease activity to warrant starting biologic medications. In this context, obesity has been associated with increased pain, disease activity, fatigue, radiographic progression, comorbidity and a lower likelihood of reduced disease activity compared with normal weight [13]. Therefore, a knowledge gap exists regarding whether BMI is associated with poor disease outcomes, exclusive of treatment response to biologics. Similarly, in psoriatic arthritis, higher body weight has been linked to worse pain, function, disease activity, joint counts, enthesitis occurrence and psoriasis scores at follow-up compared with normal weight, again in response to pharmaceutical intervention [13]. Two intervention studies in psoriatic arthritis found that weight loss improves C-reactive protein concentrations, quality of life and disease activity, although these studies were limited by relatively small sample sizes of 46 and 126 participants [22, 23]. The biological mechanisms underlying the responses to weight loss are believed to involve the reduction of pro-inflammatory cytokine production from adipose tissue, thereby mitigating the cycle of chronic inflammation, as documented in other populations with inflammatory diseases [24].

The major limitation of existing research on obesity and disease outcomes in inflammatory spondyloarthropathies is the considerable heterogeneity in study design and methodology [13]. While systematic reviews have attempted to assess the effect of body weight on disease-specific outcomes in rheumatic diseases, the evidence varies considerably among different diseases [13]. Additionally, obesity often coincides with other unhealthy lifestyle choices, such as a higher prevalence of smoking, which are known to contribute to inflammation, yet these factors are rarely controlled for [9]. Consequently, EULAR’s recommendations for weight management have been broad, covering all rheumatic diseases rather than providing disease-specific guidelines [17]. The existing guidelines have also received a grade D recommendation from EULAR, indicating inconclusive evidence [17].

The present study addresses these gaps by demonstrating that obesity is associated with significantly higher spinal and general pain, fatigue, anxiety, and mobility issues in a sample of over 1500 people with inflammatory spondyloarthritis, even after adjusting for medication. Additionally, the present study highlighted a higher prevalence of current or former smoking among individuals with obesity, but the large sample size enabled statistical correction for this to investigate the independent effect of obesity on symptom severity. This finding provides answers to one of EULAR’s 2023 research priorities, confirming that obesity is indeed linked to fatigue, independent of other lifestyle factors. Furthermore, the results also suggest that the optimal BMI for minimizing spinal pain is 22.5 kg/m^2^, a finding of clinical significance given that spinal pain is a major symptom of inflammatory spondyloarthritis.

While the current study has shown obesity to significantly impact symptom severity in individuals with inflammatory spondyloarthropathies, physical activity plays an equally important role in managing the severity of symptoms. Systematic reviews and meta-analyses have highlighted the benefits of physical activity for symptom management in inflammatory diseases such as rheumatoid and osteoarthritis [13]. However, EULAR’s 2021 guidelines on physical activity are broad, covering all rheumatic diseases as a single group [17]. Additionally, the evidence for reducing physical inactivity in these guidelines is inconsistent or inconclusive. Research specifically focusing on physical activity in individuals with inflammatory spondyloarthropathies is needed due to potential differences in how physical activity affects these conditions compared with other rheumatic diseases.

Studies have consistently demonstrated that regular physical activity is associated with improved symptom management in inflammatory diseases, with the level of evidence in rheumatoid and osteoarthritis being rated as moderate to high [13]. For instance, physical activity has been linked to reduced pain, improved physical function, and enhanced quality of life in people with these conditions [13]. In the context of inflammatory spondyloarthropathies, much of the existing research has focused on axial spondyloarthritis. Several reviews have highlighted that higher levels of physical activity and structured exercise programs (both aerobic and muscle strengthening) can improve physical function, disease activity and chest expansion, with some low-level evidence supporting improvements in pain, stiffness, mobility and cardiorespiratory function [13, 25]. A more recent randomized controlled trial also demonstrated the beneficial effects of moderate-intensity exercise on spinal pain and inflammation over 12 weeks compared with usual care, potentially due to decreases in pro-inflammatory immune cells, though this study was limited by a small sample size of 20 participants [26]. However, the effectiveness of different exercise protocols remains unclear due to the significant heterogeneity of study designs [23]. In people with psoriatic arthritis, research on the impact of physical activity on disease outcomes is limited [13]. One study demonstrated that resistance exercise performed twice a week for 12 weeks led to significant improvements in functional capacity and disease activity [21]. However, another study found that three months of high-intensity training had no definitive effect on disease activity, but did reduce fatigue [27]. Given the inconsistent and inconclusive evidence, EULAR set research priorities to investigate the influence of high levels of physical activity on pain, and the contribution of physical activity to fatigue [17, 18].

Our findings corroborate existing knowledge across populations, indicating that low physical activity contributes to a heightened risk of experiencing pain, fatigue, limited mobility and anxiety. Notably, both moderate and high levels of physical activity were associated with significant and similarly better fatigue, mobility and anxiety compared with low activity levels. Moderate levels of physical activity provided stronger benefits for pain than high activity compared with low activity, but the reasons for this remain unclear. Importantly, the benefits of physical activity were also consistent across BMI categories. This reinforces the established understanding in other populations that even moderate physical activity yields substantial symptom relief compared with low activity, regardless of BMI category [28, 29], and provides answers to EULAR’s research priority that low physical activity is associated with fatigue. Spline modelling supported the above, showing that even small amounts of physical activity were associated with large reductions in spinal pain, compared with no physical activity. However, high activity was associated with higher spinal pain compared with moderate activity, which does provide some support for the EULAR research priority to investigate the adverse effects of high amounts of exercise on pain.

The findings of this study have significant clinical implications for managing inflammatory spondyloarthropathies. Overweight and obesity are linked to higher symptom severity, including spinal pain, fatigue, anxiety, general pain and mobility issues, underscoring the need for effective weight management guidelines. Clinicians should aim for an optimal BMI of around 22.5 kg/m^2^ in patients with inflammatory spondyloarthritis to reduce spinal pain. Moderate activity is also crucial, showing strong associations with reduced symptom severity. Furthermore, those who are inactive will likely improve management of spinal pain with a small increase in physical activity levels. Therefore, specific inflammatory spondyloarthritis guidelines from NICE and EULAR should focus on weight management and physical activity for management of symptoms. While this study highlights the significant role of physical activity and weight management in managing symptoms of inflammatory spondyloathropathies, future research should also consider the potential impact of diet on symptom severity, given the relationship between a diet quality and body composition [30].

A key strength of the study is that a large sample size and comprehensive data collection allowed robust statistical methods to be used, adjusting for potential covariates. Limitations include physical activity being self-report, which is susceptible to bias, especially in those with obesity [31]. Another limitation is the large proportion of individuals who do not answer questions on pain, fatigue, mobility, and anxiety. Within the UK Biobank, only 33% of people with inflammatory spondyloarthropathies answer the questions related to symptom severity. As a result, there was a limited number of respondents with BMI vales between 18– and 20 kg/m^2^, which led to wide confidence intervals within that BMI range for spinal pain. Additionally, the use of BMI as a measure of obesity does not account for body composition, which may be particularly relevant in this population given the young age of onset and potential for high muscle mass in people with high physical activity. As a result, BMI alone may not fully capture the nuances of body composition. Further, symptoms were only assessed for a maximum period of three months leading up to the assessment day and this may skew data based on whether individuals were feeling good on the day of testing or were suffering from flares on the day of testing. This limitation could contribute to reverse causality, as it raises the question: are people with low physical activity less active due to pain, or does lower activity lead to higher pain? In patients with rheumatoid arthritis and axial spondyloarthritis, flares are reported to be frequent but generally short in duration, with persistent flares being associated with a moderate decrease in physical activity [32]. To better understand the directionality of the relationship, particularly in the context of flares, future research should employ longitudinal study designs.

In conclusion, our findings highlight the importance of weight management and physical activity promotion in the clinical management and treatment of inflammatory spondyloarthropathies. Physical activity may be beneficial even in patients who are normal weight, and weight loss (in patients with overweight or obesity) may be beneficial even in patients who are physically active. As such, our article provides the evidence necessary for NICE and EULAR to develop guidelines focusing on weight management and physical activity tailored specifically for people with inflammatory spondyloarthropathies. Future research should continue to refine these recommendations and explore the long-term benefits of lifestyle interventions in this population.

## Supplementary Material

rkae109_Supplementary_Data

## Data Availability

All data will be available upon request.
