# Supplementary material for: Combined associations of obesity and physical activity with pain, fatigue, stiffness and anxiety in adults with spondyloarthropathies: UK Biobank study
Source: Rheumatol Adv Pract. 2024 Sep 3;8(4):rkae109. doi: 10.1093/rap/rkae109 (PMC11401743; doi:10.1093/rap/rkae109)
Supplement: rkae109_Supplementary_Data [file rkae109_supplementary_data.docx]

**Supplementary Material: Combined associations of obesity and physical activity with pain, fatigue, stiffness, and anxiety in Spondyloarthropathies: UK Biobank study**

**Supplementary Table S1. Descriptive statistics for each combination of weight and physical activity status.**

|  |  | **Weight and physical activity status** | | | | | | | | |
| --- | --- | --- | --- | --- | --- | --- | --- | --- | --- | --- |
|  |  | **Normal weight, low**  **(N=91)** | **Normal weight, moderate**  **(N=204)** | **Normal weight, high**  **(N=216)** | **Overweight, low**  **(N=109)** | **Overweight, moderate**  **(N=251)** | **Overweight, high**  **(N=269)** | **Obese, low**  **(N=130)** | **Obese, moderate**  **(N=168)** | **Obese, high**  **(N=139)** |
| **Age (years)** | Median (IQR) | 56 (51,61) | 59  (51, 63) | 60  (53, 64) | 59  (53, 64) | 60  (55, 63) | 59  (53, 64) | 58  (52, 62) | 59  (53, 63) | 59  (52, 63) |
| **Disease duration (years)** | Median (IQR) | 12·9  (6·5, 24·3) | 13·8  (5·8, 27·5) | 10·2  (5·2, 21·0) | 14·9  (6·9, 30·5) | 12·5  (5·8, 25·1) | 11·5  (5·0, 21·8) | 8·8  (5·0, 20·8) | 10·5  (5·2, 17·6) | 8·9 (4·0, 19·0) |
| **Sex** |  |  |  |  |  |  |  |  |  |  |
| Male | N (%) | 36 (39·6) | 72 (35·5) | 74 (34·3) | 48 (44·0) | 128 (51·0) | 151 (56·1) | 57 (43·8) | 72 (42·9) | 64 (46·0) |
| Female | N (%) | 55 (60·4) | 132 (65·5) | 142 (65·7) | 61 (56·0) | 123 (49·0) | 118 (43·9) | 73 (56·2) | 96 (57·1) | 75 (54·0) |
|  |  |  |  |  |  |  |  |  |  |  |
| **Ethnicity** |  |  |  |  |  |  |  |  |  |  |
| White | N (%) | 83 (91·2) | 197 (96·5) | 206 (95·3) | 106 (97·2) | 245 (97·6) | 264 (98·2) | 128 (98·4) | 164 (97·7) | 137 (98·6) |
| South Asian | N (%) | 2 (2·1) | 1 (0·5) | 1 (0·5) | 2 (1·8) | 3 (1·1) | 2 (0·7) | 2 (1·6) | 2 (1·1) | 0 (0·0) |
| Black | N (%) | 1 (1·1) | 0 (0·0) | 1 (0·5) | 1 (1·0) | 2 (0·9) | 2 (0·7) | 0 (0·0) | 1 (0·6) | 1 (0·7) |
| Mixed / other | N (%) | 5 (5·6) | 7 (3·0) | 8 (3·7) | 0 (0·0) | 1 (0·4) | 1 (0·4) | 0 (0·0) | 1 (0·6) | 1 (0·7) |
|  |  |  |  |  |  |  |  |  |  |  |
| **Smoking status** |  |  |  |  |  |  |  |  |  |  |
| Never | N (%) | 46 (50·5) | 110 (53·7) | 117 (54·2) | 50 (45·9) | 132 (52·6) | 139 (51·7) | 57 (43·8) | 73 (43·5) | 59 (42·4) |
| Previous | N (%) | 32 (35·2) | 74 (36·5) | 84 (38·9) | 49 (45·0) | 99 (39·4) | 111 (41·3) | 50 (38·5) | 85 (50·6) | 73 (52·5) |
| Current | N (%) | 13 (14·3) | 20 (9·9) | 15 (6·9) | 10 (9·1) | 20 (8·0) | 19 (7·1) | 23 (17·7) | 10 (6·0) | 7 (5·0) |
|  |  |  |  |  |  |  |  |  |  |  |
| **Alcohol intake** |  |  |  |  |  |  |  |  |  |  |
| Daily or almost daily | N (%) | 22 (24·2) | 58 (28·1) | 52 (24·1) | 21 (19·3) | 56 (22·3) | 67 (24·9) | 24 (18·5) | 38 (22·6) | 24 (17·3) |
| Three or four times a week | N (%) | 14 (15·4) | 55 (27·1) | 58 (26·9) | 29 (26·6) | 55 (21·9) | 71 26·4) | 26 (20·0) | 31 (18·5) | 28 (20·1) |
| Once or twice a week | N (%) | 21 (23·1) | 50 (24·6) | 52 (24·1) | 24 (22·0) | 77 (30·7) | 62 (23·0) | 31 (23·8) | 30 (17·9) | 31 (22·3) |
| One to three times a month | N (%) | 17 (18·7) | 19 (9·4) | 21 (9·7) | 10 (9·2) | 30 (12·0) | 29 (10·8) | 10 (7·7) | 27 (16·1) | 18 (12·9) |
| Special occasions | N (%) | 11 (12·1) | 11 (5·4) | 22 (10·2) | 15 (13·8) | 20 (8·0) | 26 (9·7) | 27 (20·8) | 32 (19·0) | 26 (18·7) |
| Never | N (%) | 6 (6·6) | 11 (5·4) | 11 (5·1) | 10 (9·2) | 13 (5·2) | 14 (5·2) | 12 (9·2) | 10 (6·0) | 12 (8·6) |
|  |  |  |  |  |  |  |  |  |  |  |
| **Townsend Deprivation Index** | Median (IQR) | -2·62  (-3·87, 0·12) | -2·89  (-4·08, -0·90) | -2·57  (-3·87, -0·82) | -2·50  (-3·77, 0·31) | -2·41  (-3·88, -0·34) | -2·56  (-3·83, -0·31) | -1·63  (-3·54, 1·62) | -1·85  (-3·55, 0·40) | -1·74  (-3·44, 1·10) |
| **Medication for pain relief** |  |  |  |  |  |  |  |  |  |  |
| No medication | N (%) | 36 (39·6) | 113 (55·2) | 104 (48·1) | 50 (46·3) | 113 (45·0) | 122 (45·4) | 43 (33·1) | 55 (32·7) | 54 (38·8) |
| Pain relief and anti-inflammatory drugs (e.g., Aspirin, Ibuprofen, Paracetamol) | N (%) | 47 (51·6) | 82 (40·1) | 101 (46·3) | 51 (46·7) | 116 (46·2) | 133 (46·0) | 79 (60·7) | 99 (58·9) | 71 (51·0) |
| Gastrointestinal medication (e.g., Ranitidine and Omeprazole) | N (%) | 5 (5·5) | 6 (3·0) | 10 (4·7) | 6 (5·5) | 18 (7·2) | 13 (4·8) | 5 (3·9) | 14 (8·3) | 12 (8·6) |
| Bowel regulation (e.g., Laxatives) | N (%) | 3 (3·3) | 3 (1·5) | 1 (0·5) | 1 (0·9) | 4 (1·6) | 1 (0·4) | 3 (2·3) | 0 (0·0) | 2 (1·4) |

**Supplementary Table S2. Associations between BMI, physical activity, and the likelihood of selecting a higher severity rating for spinal pain.**

|  | **Spinal Pain^a^** | **Spinal Pain^b^** | **Spinal Pain^c^** | **Spinal Pain^d^** |
| --- | --- | --- | --- | --- |
|  | OR (95% CI) P-value | OR (95% CI) P-value | OR (95% CI) P-value | OR (95% CI) P-value |
| **Weight Status** |  |  |  |  |
| Normal weight (referent) | -- | -- | -- | -- |
| Overweight | 1·37 (1·05, 1·79) P=0·020 |  | 1·37 (1·05, 1·79) P=0·020 | 1·45 (0·78, 2·67) P=0·238 |
| Obese | 2·40 (1·80, 3·19) P<0·001 |  | 2·34 (1·76, 3·13) P<0·001 | 3·02 (1·68, 5·44) P<0·001 |
| **Physical Activity** |  |  |  |  |
| Low (referent) | -- | -- | -- | -- |
| Moderate |  | 0·58 (0·43, 0·78) P<0·001 | 0·62 (0·46, 0·83) P=0·001 | 0·77 (0·45, 1·34) P=0·358 |
| High |  | 0·69 (0·51, 0·93) P=0·014 | 0·78 (0·58, 1·05) P=0·100 | 0·83 (0·48, 1·43) P=0·503 |
| **Interaction** |  |  |  |  |
| Normal weight*low (referent) | -- | -- | -- | -- |
| Overweight*Medium |  |  |  | 0·81 (0·38, 1·70) P=0·572 |
| Overweight*High |  |  |  | 1·08 (0·52, 2·25) P=0·841 |
| Obese*Medium |  |  |  | 0·66 (0·32, 1·38) P=0·273 |
| Obese*High |  |  |  | 0·76 (0·35, 1·63) P=0·479 |

^a^Model including weight status but not physical activity. ^b^Model including physical activity but not weight status. ^c^Model including weight status and physical activity status. ^d^Model including weight status, physical activity status, and an interaction· Models were adjusted for age, sex, and ethnicity.

**Supplementary Table S3. Associations between BMI, physical activity, and the likelihood of selecting a higher severity rating for fatigue.**

|  | **Fatigue^a^** | **Fatigue^b^** | **Fatigue^c^** | **Fatigue^d^** |
| --- | --- | --- | --- | --- |
|  | OR (95% CI) P-value | OR (95% CI) P-value | OR (95% CI) P-value | OR (95% CI) P-value |
| **Weight Status** |  |  |  |  |
| Normal weight (referent) | -- | -- | -- | -- |
| Overweight | 1·46 (1·17, 1·81) P<0·001 |  | 1·48 (1·19, 1·83) P<0·001 | 1·91 (1·15, 3·18) P=0·012 |
| Obese | 2·56 (2·02, 3·24) P<0·001 |  | 2·52 (1·88, 3·20) P<0·001 | 3·10 (1·90, 5·07) P<0·001 |
| **Physical Activity** |  |  |  |  |
| Low (referent) | -- | -- | -- | -- |
| Moderate |  | 0·58 (0·44, 0·72) P<0·001 | 0·68 (0·54, 0·82) P=0·004 | 0·72 (0·46, 1·12) P=0·155 |
| High |  | 0·57 (0·44, 0·72) P<0·001 | 0·67 (0·53, 0·82) P=0·003 | 0·86 (0·55, 1·35) P=0·531 |
| **Interaction** |  |  |  |  |
| Normal weight*low (referent) | -- | -- | -- | -- |
| Overweight*Medium |  |  |  | 0·83 (0·45, 1·53) P=0·564 |
| Overweight*High |  |  |  | 0·61 (0·33, 1·13) P=0·121 |
| Obese*Medium |  |  |  | 0·86 (0·46, 1·59) P=0·643 |
| Obese*High |  |  |  | 0·61 (0·32, 1·15) P=0·129 |

^a^Model including weight status but not physical activity. ^b^Model including physical activity but not weight status. ^c^Model including weight status and physical activity status. ^d^Model including weight status, physical activity status, and an interaction· Models were adjusted for age, sex, and ethnicity.

**Supplementary Table S4. Associations between BMI, physical activity, and the likelihood of selecting a higher severity rating anxiety.**

|  | **Anxiety^a^** | **Anxiety^b^** | **Anxiety^c^** | **Anxiety^d^** |
| --- | --- | --- | --- | --- |
|  | OR (95% CI) P-value | OR (95% CI) P-value | OR (95% CI) P-value | OR (95% CI) P-value |
| **Weight Status** |  |  |  |  |
| Normal weight (referent) | -- | -- | -- | -- |
| Overweight | 1·41 (1·10, 1·82) P<0·001 |  | 1·42 (1·11, 1·83) P<0·001 | 1·53 (0·86, 2·73) P=0·149 |
| Obese | 2·16 (1·65, 2·83) P<0·001 |  | 2·40 (1·89, 3·07) P<0·001 | 2·87 (1·65, 4·99) P<0·001 |
| **Physical Activity** |  |  |  |  |
| Low (referent) | -- | -- | -- | -- |
| Moderate |  | 0·66 (0·51, 0·87) P=0·003 | 0·77 (0·61, 0·97) P=0·032 | 0·85 (0·50, 1·45) P=0·547 |
| High |  | 0·65 (0·50, 0·86) P=0·002 | 0·74 (0·59, 0·95) P=0·029 | 0·88 (0·52, 1·50) P=0·644 |
| **Interaction** |  |  |  |  |
| Normal weight*low (referent) | -- | -- | -- | -- |
| Overweight*Medium |  |  |  | 0·95 (0·47, 1·91) P=0·885 |
| Overweight*High |  |  |  | 0·87 (0·43, 1·75) P=0·696 |
| Obese*Medium |  |  |  | 0·65 (0·32, 1·31) P=0·226 |
| Obese*High |  |  |  | 0·65 (0·32, 1·32) P=0·230 |

^a^Model including weight status but not physical activity. ^b^Model including physical activity but not weight status. ^c^Model including weight status and physical activity status. ^d^Model including weight status, physical activity status, and an interaction· Models were adjusted for age, sex, and ethnicity.

**Supplementary Table S5. Associations between BMI, physical activity, and the likelihood of selecting a higher severity rating for general pain.**

|  | **General Pain^a^** | **General Pain^b^** | **General Pain^c^** | **General Pain^d^** |
| --- | --- | --- | --- | --- |
|  | OR (95% CI) P-value | OR (95% CI) P-value | OR (95% CI) P-value | OR (95% CI) P-value |
| **Weight Status** |  |  |  |  |
| Normal weight (referent) | -- | -- | -- | -- |
| Overweight | 1·36 (1·10, 1·69) P=0·005 |  | 1·42 (1·05, 1·74) P=0·032 | 1·64 (0·98, 2·76) P=0·059 |
| Obese | 2·76 (2·17, 3·50) P<0·001 |  | 3·01 (2·22, 4·65) P<0·001 | 3·69 (2·25, 6·05) P<0·001 |
| **Physical Activity** |  |  |  |  |
| Low (referent) | -- | -- | -- | -- |
| Moderate |  | 0·56 (0·44, 0·72) P<0·001 | 0·62 (0·50, 0·78) P=0·020 | 0·83 (0·53, 1·30) P=0·414 |
| High |  | 0·74 (0·58, 0·95) P=0·016 | 0·80 (0·64, 1·03) P=0·056 | 0·99 (0·63, 1·54) P=0·949 |
| **Interaction** |  |  |  |  |
| Normal weight*low (referent) | -- | -- | -- | -- |
| Overweight*Medium |  |  |  | 0·72 (0·39, 1·34) P=0·296 |
| Overweight*High |  |  |  | 0·88 (0·48, 1·34) P=0·689 |
| Obese*Medium |  |  |  | 0·59 (0·32, 1·10) P=0·098 |
| Obese*High |  |  |  | 0·76 (0·40, 1·42) P=0·384 |

^a^Model including weight status but not physical activity. ^b^Model including physical activity but not weight status. ^c^Model including weight status and physical activity status. ^d^Model including weight status, physical activity status, and an interaction· Models were adjusted for age, sex, and ethnicity.

**Supplementary Table S6. Associations between BMI, physical activity, and the likelihood of selecting a higher severity rating for mobility issues.**

|  | **Mobility^a^** | **Mobility^b^** | **Mobility^c^** | **Mobility^d^** |
| --- | --- | --- | --- | --- |
|  | OR (95% CI) P-value | OR (95% CI) P-value | OR (95% CI) P-value | OR (95% CI) P-value |
| **Weight Status** |  |  |  |  |
| Normal weight (referent) | -- | -- | -- | -- |
| Overweight | 1·78 (1·42, 2·24) P<0·001 |  | 1·98 (1·62, 2·44) P<0·001 | 2·19 (1·29, 3·72) P=0·004 |
| Obese | 5·07 (3·95, 6·49) P<0·001 |  | 5·62 (4·26, 8·14) P<0·001 | 7·46 (4·48, 12·43) P<0·001 |
| **Physical Activity** |  |  |  |  |
| Low (referent) | -- | -- | -- | -- |
| Moderate |  | 0·52 (0·41, 0·67) P<0·001 | 0·60 (0·49, 0·75) P<0·001 | 0·83 (0·51, 1·34) P=0·437 |
| High |  | 0·51 (0·40, 0·65) P<0·001 | 0·59 (0·48, 0·73) P<0·001 | 0·75 (0·47, 1·21) P=0·241 |
| **Interaction** |  |  |  |  |
| Normal weight*low (referent) | -- | -- | -- | -- |
| Overweight*Medium |  |  |  | 0·72 (0·38, 1·36) P=0·313 |
| Overweight*High |  |  |  | 0·86 (0·46, 1·61) P=0·634 |
| Obese*Medium |  |  |  | 0·52 (0·27, 0·98) P=0·042 |
| Obese*High |  |  |  | 0·63 (0·33, 1·19) P=0·156 |

^a^Model including weight status but not physical activity. ^b^Model including physical activity but not weight status. ^c^Model including weight status and physical activity status. ^d^Model including weight status, physical activity status, and an interaction· Models were adjusted for age, sex, and ethnicity. The effect of the overall interaction model was not significant.

**Supplementary Table S7. Associations between BMI, physical activity, and the likelihood of selecting a higher severity rating for spinal pain.**

|  | **Spinal Pain^a^** | **Spinal Pain^b^** | **Spinal Pain^c^** | **Spinal Pain^d^** |
| --- | --- | --- | --- | --- |
|  | OR (95% CI) P-value | OR (95% CI) P-value | OR (95% CI) P-value | OR (95% CI) P-value |
| **Weight Status** |  |  |  |  |
| Normal weight (referent) | -- | -- | -- | -- |
| Overweight | 1·36 (1·04, 1·78) P=0·023 |  | 1·36 (1·04, 1·78) P=0·023 | 1·66 (0·89, 3·12) P=0·112 |
| Obese | 2·10 (1·57, 2·82) P<0·001 |  | 2·04 (1·51, 2·72) P<0·001 | 3·03 (1·67, 5·50) P<0·001 |
| **Physical Activity** |  |  |  |  |
| Low (referent) | -- | -- | -- | -- |
| Moderate |  | 0·63 (0·47, 0·85) P=0·003 | 0·64 (0·48, 0·86) P=0·006 | 0·98 (0·56, 1·71) P=0·937 |
| High |  | 0·74 (0·54, 0·99) P=0·044 | 0·81 (0·61, 1·06) P=0·113 | 0·94 (0·54, 1·64) P=0·834 |
| **Interaction** |  |  |  |  |
| Normal weight*low (referent) | -- | -- | -- | -- |
| Overweight*Medium |  |  |  | 0·63 (0·30, 1·35) P=0·238 |
| Overweight*High |  |  |  | 0·96 (0·46, 2·04) P=0·920 |
| Obese*Medium |  |  |  | 0·54 (0·26, 1·14) P=0·106 |
| Obese*High |  |  |  | 0·67 (0·31, 1·45) P=0·306 |

^a^Model including weight status but not physical activity. ^b^Model including physical activity but not weight status. ^c^Model including weight status and physical activity status. ^d^Model including weight status, physical activity status, and an interaction. Models were adjusted for age, sex, ethnicity, alcohol intake, smoking status, medication, deprivation, and duration of condition.

**Supplementary Table S8. Associations between BMI, physical activity, and the likelihood of selecting a higher severity rating for fatigue.**

|  | **Fatigue^a^** | **Fatigue^b^** | **Fatigue^c^** | **Fatigue^d^** |
| --- | --- | --- | --- | --- |
|  | OR (95% CI) P-value | OR (95% CI) P-value | OR (95% CI) P-value | OR (95% CI) P-value |
| **Weight Status** |  |  |  |  |
| Normal weight (referent) | -- | -- | -- | -- |
| Overweight | 1·37 (1·10,1·71) P=0·004 |  | 1·38 (1·11, 1·72) P=0·004 | 2·16 (1·29, 3·60) P=0·003 |
| Obese | 2·03 (1·60, 2·60) P<0·001 |  | 1·95 (1·52, 2·49) P<0·001 | 2·94 (1·80, 4·83) P<0·001 |
| **Physical Activity** |  |  |  |  |
| Low (referent) | -- | -- | -- | -- |
| Moderate |  | 0·67 (0·52, 0·86) P=0·001 | 0·69 (0·54, 0·89) P=0·004 | 0·93 (0·59, 1·47) P=0·770 |
| High |  | 0·60 (0·47, 0·77) P<0·001 | 0·64 (0·50, 0·83) P<0·001 | 1·01 (0·65, 1·59) P=0·953 |
| **Interaction** |  |  |  |  |
| Normal weight*low (referent) | -- | -- | -- | -- |
| Overweight*Medium |  |  |  | 0·65 (0·35 1·19) P=0·163 |
| Overweight*High |  |  |  | 0·52 (0·28, 0·97) P=0·039 |
| Obese*Medium |  |  |  | 0·67 (0·36, 1·19) P=0·211 |
| Obese*High |  |  |  | 0·50 (0·27, 0·95) P=0·035 |

^a^Model including weight status but not physical activity. ^b^Model including physical activity but not weight status. ^c^Model including weight status and physical activity status. ^d^Model including weight status, physical activity status, and an interaction. Models were adjusted for age, sex, ethnicity, alcohol intake, smoking status, medication, deprivation, and duration of condition. The effect of the overall interaction model was not significant.

**Supplementary Table S9. Associations between BMI, physical activity, and the likelihood of selecting a higher severity rating anxiety.**

|  | **Anxiety^a^** | **Anxiety^b^** | **Anxiety^c^** | **Anxiety^d^** |
| --- | --- | --- | --- | --- |
|  | OR (95% CI) P-value | OR (95% CI) P-value | OR (95% CI) P-value | OR (95% CI) P-value |
| **Weight Status** |  |  |  |  |
| Normal weight (referent) | -- | -- | -- | -- |
| Overweight | 1·34 (1·04, 1·74) P=0·026 |  | 1·35 (1·04· 1·75) P=0·024 | 1·72 (0·96, 3·11) P=0·071 |
| Obese | 1·73 (1·32, 2·29) P<0·001 |  | 1·69 (1·28, 2·23) P<0·001 | 2·68 (1·53, 4·70) P<0·001 |
| **Physical Activity** |  |  |  |  |
| Low (referent) | -- | -- | -- | -- |
| Moderate |  | 0·74 (0·56, 0·97) P=0·029 | 0·76 (0·57, 1·00) P=0·049 | 1·09 (0·63, 1·88) P=0·072 |
| High |  | 0·69 (0·53, 0·92) P=0·010 | 0·73 (0·55, 0·96) P=0·026 | 1·02 (0·59, 1·76) P=0·109 |
| **Interaction** |  |  |  |  |
| Normal weight*low (referent) | -- | -- | -- | -- |
| Overweight*Medium |  |  |  | 0·73 (0·36, 1·50) P=0·394 |
| Overweight*High |  |  |  | 0·74 (0·36, 1·51) P=0·406 |
| Obese*Medium |  |  |  | 0·52 (0·26, 1·06) P=0·072 |
| Obese*High |  |  |  | 0·55 (0·27, 1·14) P=0·109 |

^a^Model including weight status but not physical activity. ^b^Model including physical activity but not weight status. ^c^Model including weight status and physical activity status. ^d^Model including weight status, physical activity status, and an interaction. Models were adjusted for age, sex, ethnicity, alcohol intake, smoking status, medication, deprivation, and duration of condition.

**Supplementary Table S10. Associations between BMI, physical activity, and the likelihood of selecting a higher severity rating for general pain.**

|  | **General Pain^a^** | **General Pain^b^** | **General Pain^c^** | **General Pain^d^** |
| --- | --- | --- | --- | --- |
|  | OR (95% CI) P-value | OR (95% CI) P-value | OR (95% CI) P-value | OR (95% CI) P-value |
| **Weight Status** |  |  |  |  |
| Normal weight (referent) | -- | -- | -- | -- |
| Overweight | 1·28 (1·03, 1·59) P=0·028 |  | 1·29 (1·03, 1·60) P=0·024 | 1·97 (1·17, 3·32) P=0·011 |
| Obese | 2·12 (1·66, 2·71) P<0·001 |  | 2·10 (1·64, 2·69) P<0·001 | 3·43 (2·09, 5·65) P<0·001 |
| **Physical Activity** |  |  |  |  |
| Low (referent) | -- | -- | -- | -- |
| Moderate |  | 0·62 (0·48, 0·80) P<0·001 | 0·66 (0·51, 0·84) P=0·001 | 1·09 (0·69, 1·72) P=0·700 |
| High |  | 0·69 (0·62, 1·02) P=0·068 | 0·87 (0·68, 1·12) P=0·286 | 1·19 (0·76, 1·86) P=0·460 |
| **Interaction** |  |  |  |  |
| Normal weight*low (referent) | -- | -- | -- | -- |
| Overweight*Medium |  |  |  | 0·51 (0·27, 0·94) P=0·032 |
| Overweight*High |  |  |  | 0·69 (0·37, 1·28) P=0·240 |
| Obese*Medium |  |  |  | 0·46 (0·25, 0·86) P=0·015 |
| Obese*High |  |  |  | 0·61 (0·32, 1·14) P=0·123 |

^a^Model including weight status but not physical activity. ^b^Model including physical activity but not weight status. ^c^Model including weight status and physical activity status. ^d^Model including weight status, physical activity status, and an interaction. Models were adjusted for age, sex, ethnicity, alcohol intake, smoking status, medication, deprivation, and duration of condition. The effect of the overall interaction model was not significant.

**Supplementary Table S11. Associations between BMI, physical activity, and the likelihood of selecting a higher severity rating for mobility issues.**

|  | **Mobility^a^** | **Mobility^b^** | **Mobility^c^** | **Mobility^d^** |
| --- | --- | --- | --- | --- |
|  | OR (95% CI) P-value | OR (95% CI) P-value | OR (95% CI) P-value | OR (95% CI) P-value |
| **Weight Status** |  |  |  |  |
| Normal weight (referent) | -- | -- | -- | -- |
| Overweight | 1·73 (1·37, 2·18) P<0·001 |  | 1·76 (1·39, 2·22) P<0·001 | 2·54 (1·49, 4·35) P<0·001 |
| Obese | 4·19 (3·25, 5·39) P<0·001 |  | 4·03 (3·13, 5·19) P<0·001 | 7·16 (4·27, 12·00) P<0·001 |
| **Physical Activity** |  |  |  |  |
| Low (referent) | -- | -- | -- | -- |
| Moderate |  | 0·56 (0·44, 0·72) P<0·001 | 0·60 (0·46, 0·77) P<0·001 | 1·03 (0·63, 1·68) P=0·907 |
| High |  | 0·52 (0·40, 0·66) P<0·001 | 0·59 (0·46, 0·76) P<0·001 | 0·84 (0·52, 1·35) P=0·468 |
| **Interaction** |  |  |  |  |
| Normal weight*low (referent) | -- | -- | -- | -- |
| Overweight*Medium |  |  |  | 0·55 (0·29, 1·04) P=0·067 |
| Overweight*High |  |  |  | 0·72 (0·38 1·37) P=0·321 |
| Obese*Medium |  |  |  | 0·42 (0·22, 0·80) P=0·008 |
| Obese*High |  |  |  | 0·53 (0·28, 1·02) P=0·056 |

^a^Model including weight status but not physical activity. ^b^Model including physical activity but not weight status. ^c^Model including weight status and physical activity status. ^d^Model including weight status, physical activity status, and an interaction. Models were adjusted for age, sex, ethnicity, alcohol intake, smoking status, medication, deprivation, and duration of condition. The effect of the overall interaction model was not significant.
